# Spontaneous bodily coordination varies across affective and intellectual child-adult interactions

**DOI:** 10.3389/fpsyg.2023.1264504

**Published:** 2024-01-16

**Authors:** Carlos Cornejo, Zamara Cuadros, David Carré, Esteban Hurtado, Himmbler Olivares

**Affiliations:** ^1^Laboratorio de Lenguaje, Interacción y Fenomenología, Escuela de Psicología, Pontificia Universidad Católica de Chile, Santiago, Chile; ^2^Universidad Icesi, Departamento de Estudios Psicológicos, Facultad de Ciencias Humanas, Cali, Colombia; ^3^Instituto de Ciencias de la Salud, Universidad de O’Higgins, Rancagua, Chile; ^4^Departamento de Psicología, Universidad de Concepción, Concepción, Chile

**Keywords:** interaction, child-adult interaction, interpersonal coordination, synchrony, contextual variation, affective interaction, intellectual interaction, types of coordination

## Abstract

Research on child-adult interactions has identified that the morphology of bodily coordination seems to be sensitive to age and type of interaction. Mirror-like imitation emerges earlier in life and is more common during emotionally laden interactions, while anatomical imitation is acquired later and associated with cognitive tasks. However, it remains unclear whether these morphologies also vary with age and type of interaction during spontaneous coordination. Here we report a motion capture study comparing the spontaneous coordination patterns of thirty-five 3-year-old (20 girls; *M*_age_ = 3.15 years) and forty 6-year-old children (20 girls; *M*_age_ = 6.13 years) interacting with unacquainted adults during two storytelling sessions. The stories narrated the search of a character for her mother (Predominantly Affective Condition) or an object (Predominantly Intellectual Condition) inside a supermarket. Results show that children of both ages consistently coordinated their spontaneous movements towards adult storytellers, both in symmetric and asymmetric ways. However, symmetric coordination was more prominent in 3-year-old children and during predominantly emotional interactions, whereas asymmetric coordination prevailed in 6-year-old children and during predominantly intellectual interactions. These results add evidence from spontaneous interactions in favor of the hypothesis that symmetric coordination is associated with affective interactions and asymmetric coordination with intellectual ones.

## 1 Introduction

Moving together has been demonstrated to be a ubiquitous feature of human interactions (Asher et al., 2020; Mayo and Gordon, 2020). Sometimes people deliberately copy the movements and gestures of others when pursuing a similar goal or when performing a joint action (Paulus, 2014). For example, people often imitate others when they are learning to play a sport, game, or activity, trying to fit into socially novel situations, or solving problems (Bernieri and Rosenthal, 1991). On other occasions, however, people spontaneously synchronize their movements during face-to-face social encounters that do not seem to require a joint action plan (Bernieri et al., 1988). This phenomenon has been called interactional synchrony or interpersonal coordination (Bernieri and Rosenthal, 1991). This phenomenon has been informed between infants and caregivers involved in turn-taking conversation contexts and daily life routines (Condon and Sander, 1974; Costantini et al., 2018; Hoch et al., 2021), as well as between couples of known and unknown adults chatting (Latif et al., 2014; Tschacher et al., 2014). Far from a coincidence, the emergence of interpersonal coordination has been associated with crucial variables for social interactions, such as prosocial attitudes and behaviors (Rennung and Göritz, 2016), like increased cooperation in 4-year-old children (Rabinowitch and Meltzoff, 2017).

Three different features of interpersonal coordination have been studied (Bernieri and Rosenthal, 1991): temporality (Nowicki et al., 2013), intensity (Latif et al., 2014), and morphology (Cuadros et al., 2019). Regarding the temporal dimension, studies have shown that motion coordination between interactants can occur both delayed in time—from milliseconds to minutes—and simultaneously, i.e., people moving together with zero lag (Cornejo et al., 2017a). A different set of studies have analyzed factors that increase or decrease the amount of spontaneous coordination between persons (Vicaria and Dickens, 2016). One of these factors is the type of interaction in which persons engage. For example, conversations exhibit higher levels of coordination when participants share a common point of view than when they disagree (Paxton and Dale, 2013; Hammal et al., 2014; Paxton and Dale, 2017). Further studies, however, have reported higher amounts of spontaneous coordination during conversations in which participants are prompted to disagree and deceive each other than during truthful ones (Duran and Fusaroli, 2017). These conflicting results point out the complex and sensitive nature of interpersonal coordination, which makes it difficult to establish a direct relation between certain types of interaction and lower or higher levels of spontaneous coordination.

Although less explored than the previous features, the morphology of interpersonal coordination has also been analyzed (Cuadros et al., 2019, 2020). This feature describes the spatial relation and the body limbs involved in the coordinated movements of interactants. For example, when two people facing each other move in the same direction on the proximity axis, both lean forward to approach the other or they lean backwards as if taking distance from each other. As this coordination behaves like being in front of a mirror, it has been referred to as mirror-like or specular coordination (Chiavarino, 2012; Sebastianutto et al., 2017). However, coordination can adopt forms diverse from the symmetric one, for instance when one interactant leans forward while the other leans backward, or when both deploy same movements but using the contralateral body limbs [one of them raises her right hand, while the other her left one]. In imitation studies, this kind of asymmetric coordination has been labeled as anatomical (Pierpaoli et al., 2014), transposed (Dunphy-Lelii, 2014) or contralateral (Erjavec and Horne, 2008). Nonetheless, asymmetric coordination has also been found studying spontaneous interactions [e.g., complementary coordination (Cornejo et al., 2018)]. In such cases, the morphology of coordination seems to be sensitive to the nature of the interaction. For example, it has been shown that the morphology of coordinated movement changes after a breach of trust during a conversation (Cornejo et al., 2018). Yet, we still do not know much about how these morphological patterns vary across different kinds of spontaneous interactions.

By contrast, the role of morphology has been more extensively explored in goal-directed imitation research (Wapner and Cirillo, 1968; Gleissner et al., 2000; Dunphy-Lelii, 2014; Sebastianutto et al., 2017). It is important to notice that, in this field, imitation is explicitly prompted by asking participants (usually children) to reproduce a model’s movement (e.g., “Do what I do”). Nonetheless, imitation studies have reported that adults and children alike exhibit the spontaneous tendency to imitate symmetrically rather than asymmetrically (i.e., “mirror-like style” rather than “anatomically”) (Erjavec and Horne, 2008; Ubaldi et al., 2015). This preference for mirror-like imitation has been associated with the establishment and maintenance of affective bonds (Dunphy-Lelii, 2014), since it emerges rapidly and spontaneously from a very early age and has been reported to be impaired in people diagnosed with ASD (Avikainen et al., 2003). Anatomical imitation, on the other hand, has been associated with intellectual situations, since difficulties to imitate anatomically decrease along with age or with simplified instructions (Bekkering et al., 2000). In particular, anatomical imitation has been reported to become less challenging for 4- to 6-years-old children with high scores in perspective-taking tasks (Dunphy-Lelii, 2014; Pierpaoli et al., 2014). Therefore, evidence from goal-directed imitation studies suggest that each morphological pattern is related to different situations and also varies with age (Wapner and Cirillo, 1968; Dunphy-Lelii, 2014; Pierpaoli et al., 2014). Mirror-like morphological patterns predominantly emerge in emotionally laden interactions from a very early age. Anatomical patterns are more prominent in predominantly intellectual interactions from 6 years of age onwards.

A recent study exploring spontaneous children-adult interactions found that 3-year-old children spontaneously coordinate both in symmetric and asymmetric ways in conversational settings (Cuadros et al., 2021). Symmetric patterns predominantly emerged in emotionally laden interactions, and asymmetric ones were more prominent in interactions centered on spatial and intellectual tasks. This finding establishes similarities and differences between the morphology of spontaneous interactions and that described in instruction-guided imitations. On the one hand, asymmetric morphology is observed in spontaneous interactions at an age reported as challenging for goal-directed imitation. On the other hand, the associations between morphological patterns and types of interactions found at age 3 years replicate those previously identified by the literature on goal-directed imitation from 6 years of age onwards. Although each morphological pattern of spontaneous coordination seems to be related to different situations, it is still unclear if they vary with age. Therefore, the present study aims to explore spontaneous coordination in conversational settings in two age groups: 3- and 6-year-old children.

To do so we conducted a study comparing the spontaneous coordination patterns displayed by 3-year-old and 6-year-old children interacting with unacquainted adults during interactions predominantly affective or intellectual. In the Predominantly Affective Interaction (PAI), the unfamiliar adult told the story of a character searching and finding her mother inside a supermarket. In the Predominantly Intellectual Interaction (PII), the unfamiliar adult narrated a story about searching and finding an object inside a supermarket. In the first condition (PAI), the story involves a spatial search with a stronger emotional engagement. In the second condition (PII), the story implies a spatial search based on information about the place and direction of objects in space. Following the literature on goal-directed imitation (Gleissner et al., 2000; Erjavec and Horne, 2008; Dunphy-Lelii, 2014; Pierpaoli et al., 2014; Ubaldi et al., 2015), we expected differences on the morphology of coordinated movement by age and condition. Given the evidence on the reported difficulties of young children to imitate anatomically, we anticipated that symmetrical coordination would be more prominent in 3-year-old children. In contrast, we expected asymmetrical coordination to be predominant in 6-year-old children. Regarding differences by condition, we expected symmetrical coordination to prevail in the emotional condition, as it has been suggested that mirror-like imitation is associated with affectively-laden situations. In contrast, we anticipated asymmetrical coordination to be more prominent in the predominantly intellectual condition (PII) since imitation studies have shown associations between anatomical imitation and intellectually-oriented situations. We used an optical motion capture system (henceforth: mocap) to accurately track and compare participants’ movements.

## 2 Materials and methods

### 2.1 Participants

Thirty-five 3-year-old (20 girls; *M*_age_ = 3.15 years, range: 3.01–3.30 years) and forty 6-year-old children (20 girls; *M*_age_ = 6.13 years, range: 6.01–6.25 years), all typically developing, were recruited from day nurseries at the Chilean National Board of Kindergartens. This sample size provided enough degrees of freedom for each group (>17,000) to assure high statistical power. The number of degrees of freedom, as dictated by the Fisher transform technique for assessing the signification of correlations, is based on the number of number pairs in pooled correlations (equivalent to the number of frames in our motion capture recordings). Fourteen additional children were excluded from the analysis due to technical issues during movement recording. The study followed the guidelines of the Declaration of Helsinki and was approved by the Social Sciences and Humanities ethics committee at the P. Universidad Católica de Chile; informed consent was obtained from all parents.

### 2.2 Design

Participants took part in an individual storytelling session with one of two female storytellers (*M*_age_ = 23.9 years; *SD* = 1.5 years), who were blind to the objectives of the study and counter-balanced for each group. Children were divided by age into two groups (3 and 6 years of age) and then randomly assigned to one of two conditions: either the Predominantly Intellectual Interaction (PII) (*n_3-year-old_* = 18, 10 girls; *n_6-year-old_* = 21, 11 girls); or the Predominantly Affective Interaction (PAI) (*n_3-year-old_* = 17, 10 girls; *n_6-year-old_* = 19, 9 girls). We chose storytelling since it is a familiar, engaging activity for children of these ages (Russell and van den Broek, 1988; Catala et al., 2017). In addition, developmental psychology research has reported that storytelling involves cognitive, social, and emotional levels (Kim, 1996; Garzotto, 2014; Cremin et al., 2017). To control for potential differences in emotions before storytelling, all parents reported the perceived children’s affect states by answering the Spanish version of the PANAS-C-SF (Sanmartín et al., 2018). Finally, interpersonal coordination between children and storytellers was measured using mocap.

### 2.3 Apparatus

**Figure 1:**
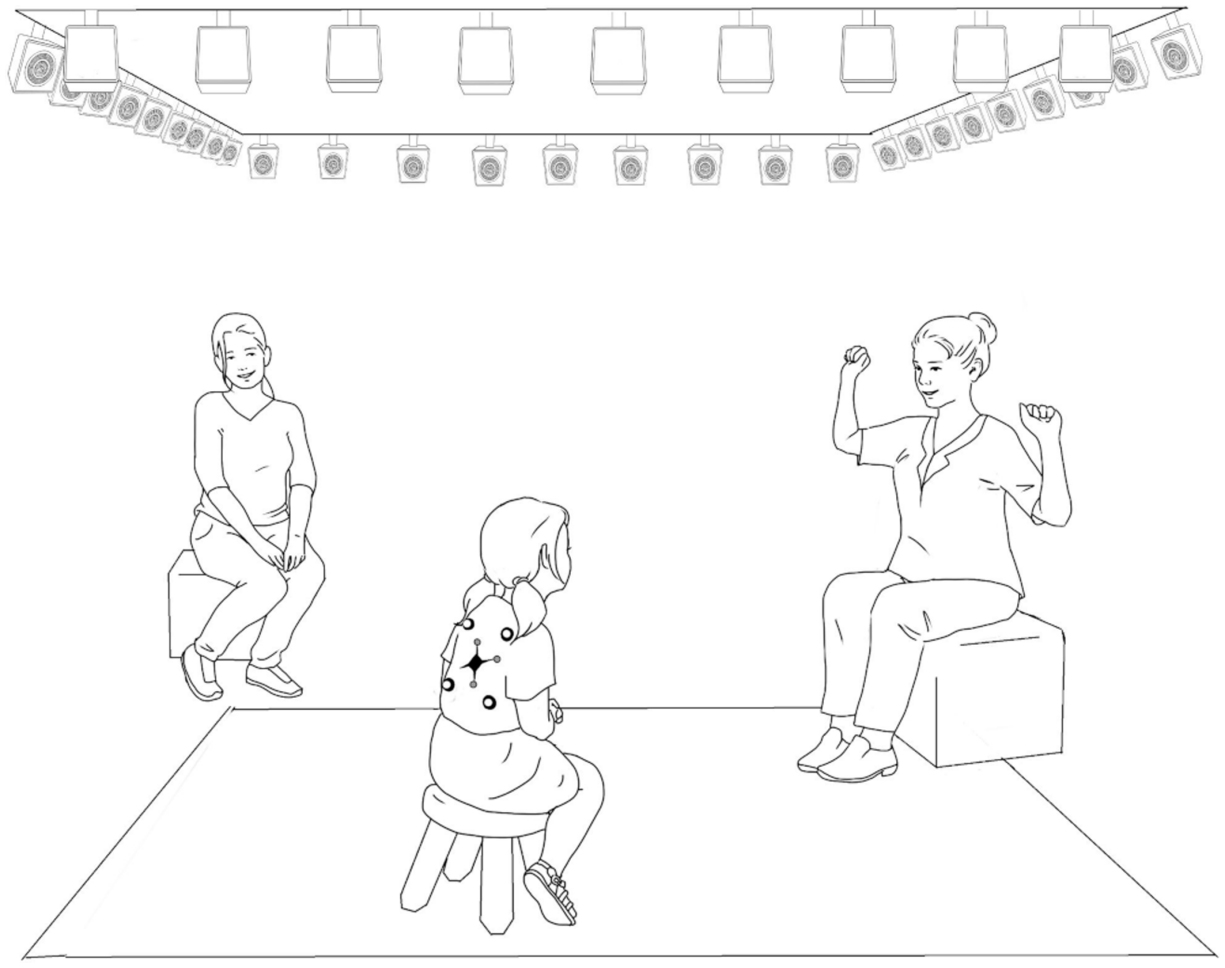
Experimental setup. Depiction of the experimental setup. The storyteller sat in front of children, while parents sat perpendicular from them. Seven reflective markers were placed in the back of children and storytellers as depicted: two in the lower-back area, two in the upper-back area, and a three-marker identifier at the center. The camera array was placed around the setting, hanging from the ceiling.

Movements above 0.1 mm were recorded at 120fps using a *NaturalPoint Optitrak* optical motion capture system equipped with thirty-six *Prime41* infrared cameras (see Figure 1). Seven reflective markers (1 cm of diameter, <10gr of weight) were used to register the torso movements of children and storytellers (see Figure 1). Data was registered using *Motive 2.2*, special software provided by the system manufacturer that calibrated, synchronized and combined the input of the camera array.

### 2.4 Procedure

Upon participants’ arrival, a research assistant showed the mocap setup functioning along the storyteller, who was already wearing a lab coat with reflective markers attached. Children were invited to participate in a mocap demonstration with their parents, who were asked to put a lab coat with reflective markers on their child. Parents were asked to report the emotional states they had so far perceived in their child at the laboratory by answering the PANAS-C-SF (Sanmartín et al., 2018). After this, the assistant invited the children to free play with her using Lego blocks while the storyteller remained next to the assistant.

After this warm-up, the storyteller narrated one of two stories depending on the condition assigned. Both stories described how the storyteller looked for something inside a supermarket, but differed on their climax, i.e., when the storyteller revealed what was lost. While in the Predominantly Intellectual Interaction (PII) condition the storyteller was looking for bread, in the Predominantly Affective Interaction (PAI) condition she was looking for her mother. By changing the object of the search, we intended to generate different degrees of affective involvement in both conditions. Storytellers were instructed to narrate the PII story with flat affection and the PAI more expressively, using facial gestures and adding vocal emphases. In both stories storytellers were asked to move their torso identically, following the spatial direction of the narrated actions (e.g., moving forward, going backwards, turning left or right). All children were invited to listen to the story as carefully as possible and no instructions were given about how they should move.

Interactions based on PII condition lasted on average 5 min and 21 s (*SD* = 33 s) for the 3-year-old group, and 5 min and 2 s for the 6-year-old group (*SD = 22 s*). Narrations of the PAI condition lasted on average 5 min and 6 s (*SD* = 15 s) for the 3-year-old group, and 5 min and 3 s for the 6-year-old group (*SD = 10s*). No statistically significant difference in the mean interaction time between cells is observed (chi-squared (1) = 0.1103, *p* = 0.7398).

Children’s initial affective states did not differ across conditions and age, neither for positive (condition: *F*(1, 73) = 2.956, *p* = 0.09; age: *F*(1, 73) = 0.527, *p* = 0.47) nor negative (condition: *F*(1, 73) = 0.108, *p* = 0.74; age: F(1, 73) = 0.014, *p* = 0.91) affect.

### 2.5 Data analysis

#### 2.5.1 Preprocessing

Raw movement data was processed with *CuteDots*, a set of custom Python scripts (available at https://github.com/estebanhurtado/cutedots) designed for labeling and visualization of mocap markers. Each marker was labeled for interactant (child, storyteller) and body part (left/right upper back, left/right lower back) to compare their movements as a pair of time series.

#### 2.5.2 Computation of cross-correlations

Cross-correlation coefficients were used to calculate coordination between children’s and storytellers’ movement. First, for each interactant we averaged upper and lower back markers into a single 3D position, only keeping 1D positions in the direction lying between the interactants (i.e., proximity axis). Second, at each frame we subtracted the position of each marker from the immediately previous frame to compute discrete speed signals (distance over time). Unlike position, which is based on an arbitrary origin point, speed usually has a zero mean from which motion events depart and return, making speed signals appropriate for correlation analysis. Third, to remove recording artifacts, we applied a single pole 10 Hz cutoff frequency low-pass filter. Fourth, we computed cross-correlation coefficients (Pearson scale) in time domain for each pair of speed time series ( a,b ):


(1) $$r_{\mathrm{ab}}=\frac{\sum_{i=1}^{n}a_{i}b_{i}\;}{\sqrt{\sum_{i=1}^{n}a_{i}^{2}}\sqrt{\sum_{i=1}^{n}b_{i}^{2}}}$$

This calculation informs the presence of similar motion occurring at zero-lag along the interaction. Positive coefficients indicate the consistent presence of mirror-like coordination. Negative coefficients report the consistent presence of coordinated movements occurring in opposite directions, e.g., when one interactant leans backward the other simultaneously leans forward. Coefficients around zero stand for no sustained coordination.

To find non-simultaneous coordinations, we computed 15 additional correlations at consecutive time delays of 100 ms (from 100 to 1,500 ms) by fixing the storyteller’s time series while progressively offsetting the children’s one, trimming signal ends that did not overlap. These delayed correlations informed if children moved as the storyteller had done, for example, 300 milliseconds before.

#### 2.5.3 Aggregation of data

To obtain an aggregated cross-correlation curve integrating information from all interactions within a group, we took each Pearson correlation to a normal distribution by means of a Fisher transform:


(2) $$x_{i}=\frac{1}{2}\ln\frac{1+r_{i}}{1-r_{i}}=\tanh^{-1}\;r_{i}$$

Here, $r_{i}$ is a Pearson correlation at some given lag time for couple i , and $x_{i}$ is the transformed variable. Standard error for $x_{i}$ is $\sigma_{i}\approx \frac{1}{\sqrt{m_{i}}}$ , where $m_{i}$ is the number of pairs involved in the calculation of correlation $r_{i}$ . To compute a group grand average, we averaged all $x_{i}$ values for the same lag time. Then we repeated the same calculation for all time lags to obtain a grand average curve per group.

From the previous standard error formula, variance for each $x_{i}$ is $\sigma_{i}^{2}\approx \frac{1}{m_{i}}$ . If we assume similar values for all $m_{i}$ (correlated signal lengths), the average of n values has a standard error of


(3) $$\sigma \approx \frac{1}{n}\sqrt{\overset{n}{\underset{i}{\sum }}\frac{1}{m_{i}}}\approx \frac{1}{\sqrt{\sum_{i=1}^{n}m_{i}}}$$

Therefore, the standard error of the grand average corresponds to the standard error of a single Fisher-transformed correlation for a signal length equivalent to the sum of all individual couple signal lengths. Consequently, a grand average X of several $x_{i}$ values can be transformed back to an aggregated Pearson correlation R by applying the inverse Fisher transform,


(4) R=tanhX

By computing these aggregated correlations for all lag times, we obtained an aggregated cross-correlation curve for each group.

#### 2.5.4 Statistical inference

The use of a normally distributed X value with known standard allowed us to perform statistical inference, based on z-tests, to test whether correlation coefficients of any group deviated from zero more than it would be expected by chance. We calculated a confidence interval with α=0.001 and Bonferroni correction (41 comparisons, corrected *p* = 0.00002) around the X value by multiplying the standard error from Eq. 3 by a cut off value from z-distribution. Then we converted the upper and lower limits of the interval back to Pearson correlation using the inverse Fisher transform in Eq. 4. These confidence intervals are shown in our plots as transparent overlays.

#### 2.5.5 Within-dyad approach

To control for effects unrelated to our experimental manipulation, we implemented a within-dyad (as in within-subject) approach. If the manipulation worked as expected, there should be a difference between coordination patterns before and after the stories’ climax, i.e., when it was revealed what had been lost (bread or the storyteller’s mother). Thus we set pre-climax coordinations as baseline and subtracted them from post-climax coordinations (see Supplementary Figure S1) using Fisher-transformed, normally distributed values. The resulting standardized cross-correlations coefficients should be interpreted as comparative rather than absolute levels of coordinated movement, since they represent the change created by the narrative climax.

#### 2.5.6 Data visualization

**Figure 2:**
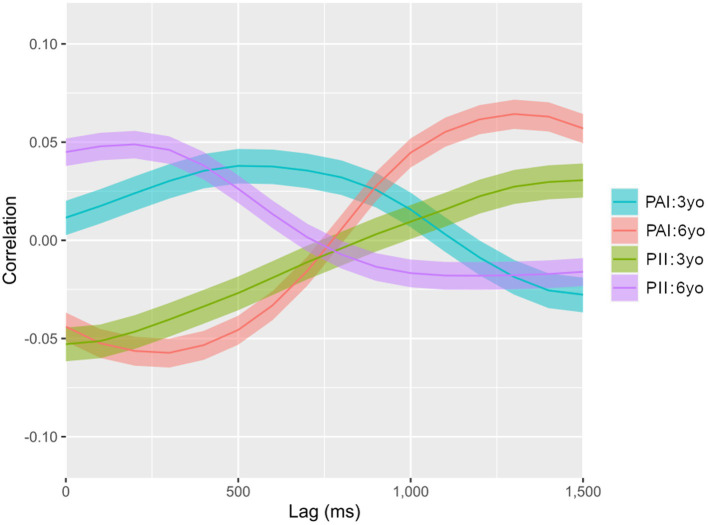
Levels of simultaneous and delayed coordination for each group. Levels of cross-correlation between speed signals of interactants’ torso movements, obtained by subtracting the post-climax movement data from the pre-climax. Solid lines display correlation coefficients, while transparent overlays show the limits of the 99.99% confidence interval for each coefficient. There are correlations not attributable to chance wherever overlays do not touch the zero correlation value. There are differences between groups wherever curves do not overlap their confidence intervals. PAI, Predominantly affective interaction; PII, Predominantly intellectual interaction.

Given their consecutive order, correlation coefficients were plotted into a single curve per group (see Figure 2). The vertical axis shows the magnitude of the correlation (in Pearson scale) as well as the predominant morphology of coordinated movement: positive values (upper half of the graph) account for symmetric coordination; negative ones (lower half of the graph) represent asymmetric coordination. The observed magnitudes are similar to those reported by previous studies assessing coordinated motion in natural-like situations (Ramseyer and Tschacher, 2011; Paxton and Dale, 2013; Latif et al., 2014). The horizontal axis corresponds to time delays. Correlations at 0 ms account for simultaneous coordination between children and storytellers. Correlations at the right of 0 ms account for children’s movements similar to the storyteller’s after a given delay (from 100 ms to 1,500 ms).

## 3 Results

The levels of simultaneous and delayed coordination between children and storytellers for each age group and condition are presented in Figure 2.

As Figure 2 shows, all groups except for the 3-year-old PAI group presented coordination at zero-lag, with similar magnitudes. The 3-year-old PII group, however, is the only one that had its peak of coordination at zero-lag with asymmetric morphology ( R = −0.05). The 3-year-old emotional group instead had a peak ( R = 0.04) of symmetric coordination at 500 ms of delay. Similarly, the 6-year-old PII group had a peak ( R = 0.05) of symmetric coordination around 250 ms of delay. In its turn, the 6-year-old PAI group had two peaks of coordinated movement: asymmetric at 250 ms ( R = −0.05) and symmetric at 1300 ms ( R = 0.06) of delay.

To some extent, all groups presented this inversion of the predominant coordination pattern at a certain time-delay (i.e., transecting the zero correlation value). This means that groups having more symmetric coordination around zero-lag (3-year-old PAI, 6-year-old PII), displayed more asymmetric patterns for delayed coordinations. Conversely, the 3-year-old PII and 6-year-old PAI groups presented more asymmetric patterns around zero-lag and more symmetric coordinations for delayed coordinations. Therefore, depending on the time scale observed, the same group presented different predominant coordination patterns.

**Figure 3:**
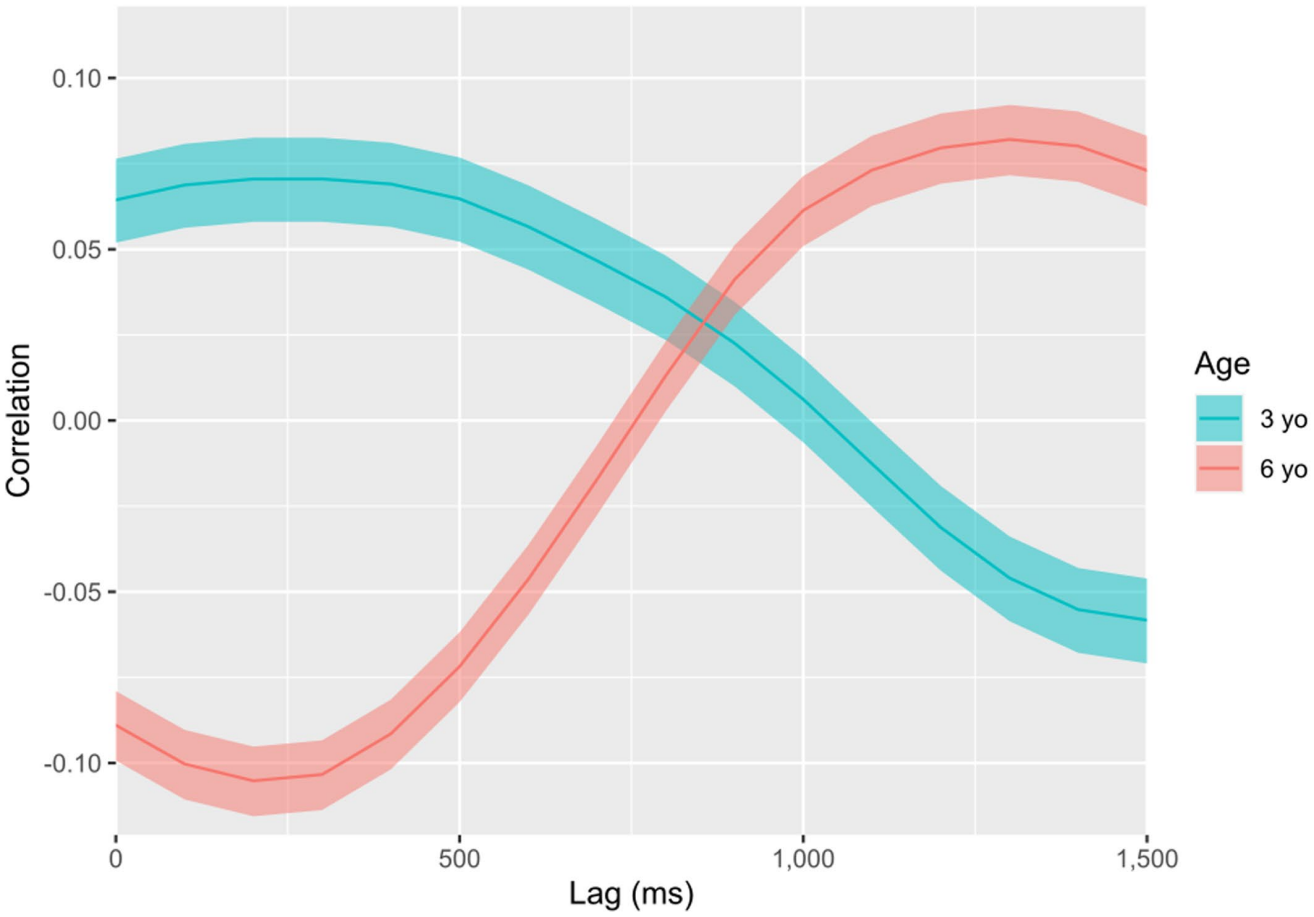
Levels of simultaneous and delayed coordination by age group. Levels of cross-correlation between speed signals of interactants’ torso movements along the interaction, obtained by subtracting the PAI (predominantly affective interaction) condition movement data from the PII (predominantly intellectual interaction) condition. Solid lines display correlation coefficients, while transparent overlays show the limits of the 99.99% confidence interval for each coefficient. There are correlations not attributable to chance wherever overlays do not touch the zero correlation value. There are differences between groups wherever curves do not overlap their confidence intervals.

**Figure 4:**
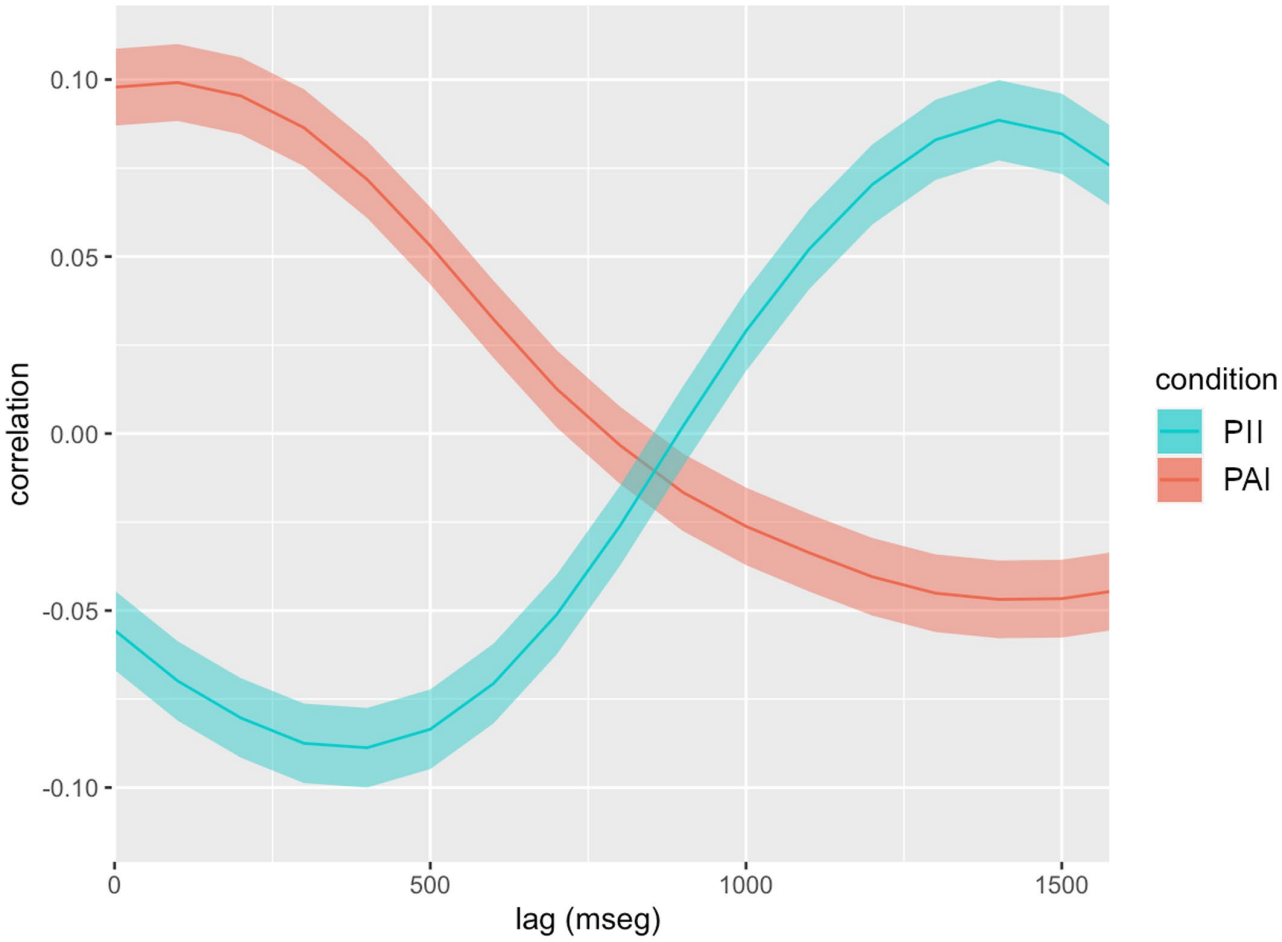
Levels of simultaneous and delayed coordination by condition. Levels of cross-correlation between speed signals of interactants’ torso movements along the interaction, obtained by subtracting the 6-year-old group movement data from the 3-year-old group. Solid lines display correlation coefficients, while transparent overlays show the limits of the 99.99% confidence interval for each coefficient. There are correlations not attributable to chance wherever overlays do not touch the zero correlation value. There are differences between groups wherever curves do not overlap their confidence intervals. PAI, Predominantly affective interaction; PII, Predominantly intellectual interaction.

Due to the unclear effects observed, we decided to simplify our grouping approach by looking for the predominant patterns of coordination by age and by condition separately. To isolate these effects, we repeated the subtraction process described above (see *Within-dyad approach*), setting the 3-year-old group as the baseline for age, and the PII group as the baseline for condition. As noted, this subtraction approach helped us to find effects that could be attributable to differences between the groups, avoiding eventually confounding averages. By doing so, we obtained a separate visualization of the effect of age (Figure 3) and condition (Figure 4) over coordination.

Thus, early coordinations (around 250 ms) were more symmetric in 3 year olds ( R = 0.07), but more asymmetric in 6 year olds ( R = −0.11). This pattern reverses for delays bigger than 700 ms, so that the 3-year-old group displayed a peak of asymmetric coordination at 1500 ms ( R = −0.06), whilst the 6-year-old group a peak of symmetric coordination at 1300 ms ( R = −0.08).

Predominantly Intellectual Interactions (PII) showed a peak of asymmetric coordination around 400 ms ( R = −0.08) but also a peak of symmetric coordination ( R = 0.09) at 140 ms of delay. Comparative rather than absolute levels of coordinated movement, since they represent the change created by the narrative climax. Conversely, the Predominantly Affective Interactions (PAI) had a peak of early symmetric coordination ( R = 0.10) around 100 ms and a peak of asymmetric coordination ( R = −0.05) around 1,400 ms. Therefore, the effect of condition over coordination also varied according to the time scale observed.

## 4 Discussion

The present study aimed to explore spontaneous coordination patterns in 3-year-old and 6-year-old children interacting with unacquainted adults. Our results show that children of both ages spontaneously coordinated their movements towards adult storytellers in symmetric and asymmetric ways. However, symmetric coordination was more prominent in 3-year-old children, whereas asymmetric coordination was more predominant in 6-year-old children. Furthermore, while symmetric imitation prevailed in the predominantly affective interaction condition (PAI), asymmetric coordination was prevalent in the predominantly intellectual interaction condition (PII). These results add evidence in favor of the hypothesis that symmetric coordination appears in interactions affectively guided, whilst asymmetric imitation in intellectually guided ones. In general terms, our findings are in line with previous works on goal-directed imitation functions (Avikainen et al., 2003; Dunphy-Lelii, 2014; Pierpaoli et al., 2014). However, they extend them since in our study symmetric and asymmetric coordination patterns were tracked during spontaneous social interactions, in children of two different age groups, and during two types of interactions. Furthermore, the spontaneous coordination patterns displayed by children were more complex than those previously observed in imitation tasks where coordination is deliberately induced.

In line with goal-directed imitation research, our results confirm the hypothesis that coordination patterns change with age—with symmetric coordination being comparatively more prominent in 3-year-old children and asymmetric coordination comparatively more prominent in 6-year-old children. However, as a recent study reported (Cuadros et al., 2021), this pattern only sustained for early coordinations—in our data, from zero-lag to 800 ms of delay. For this tendency reverses in both age groups for coordinations occurring between 1,000 and 1,500 ms of delay. Thus 3-year-old children coordinate in symmetric fashion around zero-lag, but they coordinate asymmetrically after about 1 second of delay. Conversely, early coordinations of 6-year-old children are asymmetric, but after 1 second of delay they tend to coordinate symmetrically. These findings are consistent with the hypothesis that 3-year-old children approach activities from an affective disposition, while 6-year-olds tend to approach social encounters with strangers with a focus on the intellectual task at hand (Dunphy-Lelii, 2014; Pierpaoli et al., 2014).

The inversion of spontaneous coordination patterns that we observed differs from previously reported imitation patterns. To better understand this difference, it should be noted that while imitation studies prompt children’s movements by providing explicit instructions, we studied spontaneous child-adult coordination. Thus, although goal-directed imitation and spontaneous coordination roughly belong to the same family of movements, they cannot be considered as one and the same phenomenon. Imitation studies base their conclusions on children’s capacity to follow explicit requests of imitating the movements of an adult model. On the other hand, spontaneous interpersonal coordination corresponds to the coincidence of movements between two or more people engaged in situations where movement is not scripted. Therefore, while imitation studies take a snapshot of the immediate child’s movement after the model, interpersonal coordination research—like the present study—registers the dynamic of children’s spontaneous movement along the interaction.

Our results also suggest that differences in coordination patterns between 3-year-old and 6-year-old children do not rest on their capacity to imitate or coordinate in a specific modality, since both groups exhibit the two types of coordination. The data rather show that in both age groups one modality is just more predominant than the other, namely symmetric in 3-year-old and asymmetric in 6-year-old children. This non-discrete approach would explain why, in more spontaneous interactions, 3-year-old children do not manifest the difficulties to imitate asymmetrically that has been reported in goal-directed imitation research. This approach would instead point out that the flow of the interaction changes along its course, emphasizing the variability of coordination. These fluctuations should not be unexpected since natural human movements are continuously co-adapting to those of others during social interactions (Boker et al., 2002; Ramseyer and Tschacher, 2011; Cornejo et al., 2017b). Evidence from studies tracking movements in ecologically-sound settings has reported changes in coordination patterns between adults and in infant-adult interactions within brief spans of time (between 800 ms and 1,000 ms) similar to those found in this study (Cornejo et al., 2018).

The present study also provides novel insight on how morphology varies across different types of interaction—regardless of age. Previous research has found higher interpersonal spontaneous coordination when people interact in affiliative than argumentative contexts (Paxton and Dale, 2013; Paxton and Dale, 2017), but it also has found even more coordination during deceitful interactions (Duran and Fusaroli, 2017). While seminal in assessing contextual variations, these studies only address one of the features of coordination: intensity. By means of a finer data collection technology, our study also describes qualitatively different morphologies and temporalities of coordination across different types of interaction (predominantly affective and predominantly intellectual). Thus, we found that in emotionally-laden interactions an initial symmetric coordination (from zero-lag to 800 ms of delay) was followed by asymmetric coordination (from 1,000 to 1,500 ms of delay), while in intellectually driven interactions we detected the inverted pattern. These results partially support the findings of previous imitation studies, which associated mirror-like patterns with affectively-laden situations and anatomical patterns with intellectual ones. Our data confirm this association, but only for spontaneous coordinations occurring with less than 800 ms of delay. The inversion of morphological patterns for each type of interaction observed after 1,000 ms of delay could be interpreted as another sign of the fluid and continuous nature of people’s spontaneous movements during social interactions.

Additionally, existing research on contextual variations of spontaneous coordination has relied on broad experimental manipulations, like asking participants to deceive their interaction partner (Brambilla et al., 2016; Duran and Fusaroli, 2017), in order to create different types of interaction. Our study complements this approach by taking a different route: introducing differences in the content of the stories narrated. Interestingly, this change—in comparison to previous studies—produced sustained differences both in the morphology and temporality of coordination patterns displayed by both age groups. Moreover, the manipulation used in this study cannot be adequately described in terms of contextual constraints (Paxton and Dale, 2017) or functional specificity (Duran and Fusaroli, 2017). Both physical constraints and the social function of the interaction were tantamount in both conditions, consisting in storytelling by an adult to a child. Therefore, the differences observed should not be explained by appealing to physical features of the interaction, but rather to the differing qualitative engagement produced in the predominantly affective and predominantly intellectual conditions. The difference between them could be explained just as differences in semantic content. But it is much better understood when looking at the meaning that those differences could have had for participants, since looking for bread has quite different implications than looking for someone’s mother.

These results support a vision of coordination as an encounter between people, where interactants are continuously co-adapting their movements, reciprocally acting and reacting. Our evidence suggests that conceiving coordination as a mere imitative reaction to an observed other reduces the complexity of human interaction. Similarly, these results show that human coordination, far from being a mechanical coupling between two physical systems, is highly sensitive to the quality of the interaction where it emerges (Cornejo et al., 2017b). Thus we observed different patterns of coordination depending on the type of interaction in which they engage, the age of the interactants, and probably other variables we still do not know.

Finally, we would like to point out some limitations of this study and how they could be addressed in future research. A limitation of the present study is comparing only two age groups. While doing so is useful to detect potential differences, it does not inform us about the process behind the change in coordination patterns observed. Future studies should take this into account by comparing movement data from children that are closer in age. Likewise, future research on the topic should study how children’s spontaneous coordination patterns emerge during interactions with peers and different kinds of acquainted adults (e.g., parents, family members, and teachers). Furthermore, it should be noticed that, for the sake of naturality, we did not control the storytellers’ expressiveness of gestures and pitches. Although expressiveness in storytelling must exhibit consistency with the story content to be believable in natural settings, future research might differentiate the effects on coordination of the story content and the expressivity of its telling. Besides, while we measure affect states prior to interactions as a control, it would be relevant to also assess them just as interaction, i.e., continuously during the interaction; particularly if one of the experimental conditions aims to create an emotional situation. Finally, although our analytical approach lets us show differences in movement coordination between storytellers and children depending on the child’s age (3 or 6 years old) and the type of story (affective or intellectual), it does not cover other variables that could be involved (such as the disparate competencies between children and adults in controlling their bodies). Future studies could benefit from additional analysis or different perspectives to further explore the multivariate dimension and the complexity of bodily motion between adults and children.

## Data availability statement

The raw data supporting the conclusions of this article will be made available by the authors, without undue reservation.

## Ethics statement

The studies involving humans were approved by the Social Sciences and Humanities Ethics Committee at the Pontificia Universidad Católica de Chile. The studies were conducted in accordance with the local legislation and institutional requirements. Written informed consent for participation in this study was provided by the participants' legal guardians/next of kin.

## Author contributions

CC: Conceptualization, Funding acquisition, Investigation, Methodology, Project administration, Supervision, Writing – original draft, Writing – review & editing. ZC: Conceptualization, Data curation, Formal analysis, Investigation, Methodology, Supervision, Validation, Writing – original draft, Writing – review & editing. DC: Conceptualization, Data curation, Formal analysis, Funding acquisition, Investigation, Methodology, Software, Writing – original draft, Writing – review & editing. EH: Conceptualization, Data curation, Formal analysis, Investigation, Methodology, Software, Visualization, Writing – review & editing. HO: Conceptualization, Data curation, Investigation, Resources, Writing – review & editing.
