# Supplementary material for: Spontaneous bodily coordination varies across affective and intellectual child-adult interactions
Source: Front Psychol. 2024 Jan 16;14:1264504. doi: 10.3389/fpsyg.2023.1264504 (PMC10824830; doi:10.3389/fpsyg.2023.1264504)
Supplement: Supplementary file 1 [file Table_1.DOCX]

Supplementary Material

# Supplementary Figures and Tables

## Supplementary Figures


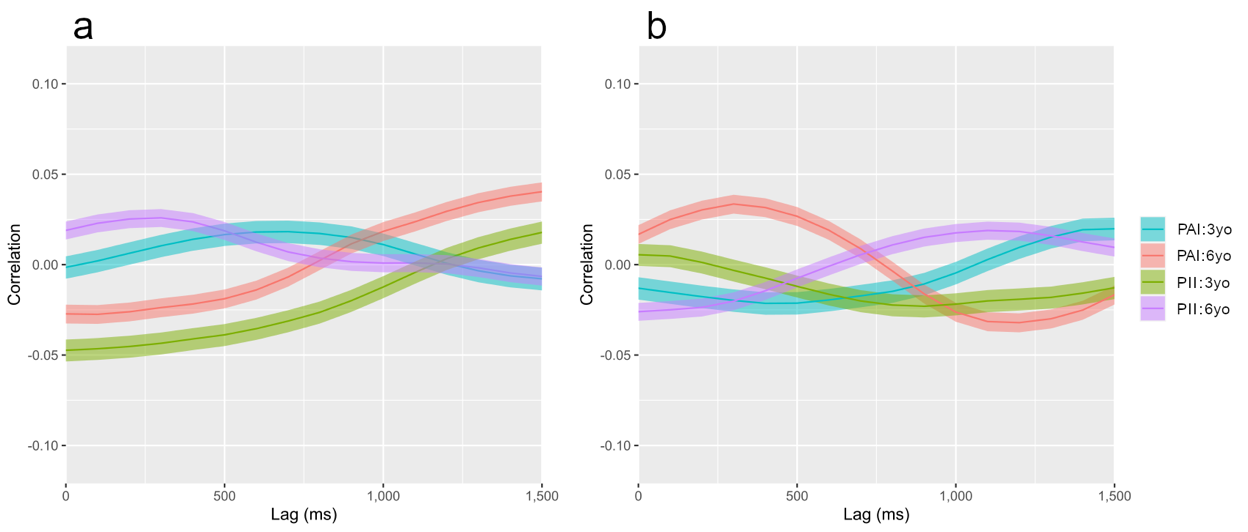


**S1 Fig. Coordination curves before and after narrative climax.** The figures presented display the pre- and post-climax correlations of each group used for computing within-dyad subtractions. a. Pre-climax correlations. b. Post-climax correlations. PAI: Predominantly affective interaction; PII: Predominantly intellectual interaction.
